# Characteristics of Plasmacytoid Dendritic Cell and CD4+ T Cell in HIV Elite Controllers

**DOI:** 10.1155/2012/869505

**Published:** 2012-11-21

**Authors:** Jean-Philippe Herbeuval, Nikaïa Smith, Jacques Thèze

**Affiliations:** ^1^CNRS UMR 8147 and Hôpital Necker, Université Paris Descartes, 149-161 rue de Sèvres, 75015 Paris, France; ^2^CNRS UMR 8601, Université Paris Descartes, 45 rue des Saint-Pères, 75006 Paris, France; ^3^Unité d'Immunologie Humaine, Centre Medical Necker Pasteur, Institut Pasteur, 28 Rue du Docteur Roux, 75015 Paris, France

## Abstract

Despite variability, the majority of HIV-1-infected individuals progress to AIDS characterized by high viral load and massive CD4+ T-cell depletion. However, there is a subset of HIV-1-positive individuals that does not progress and spontaneously maintains an undetectable viral load. This infrequent patient population is defined as HIV-1 controllers (HIV controllers), and represents less than 1% of HIV-1-infected patients. HIV-1-specific CD4+ T cells and the pool of central memory CD4+ T cells are also preserved despite immune activation due to HIV-1 infection. The majority of HIV controllers are also defined by the absence of massive CD4+ T-cell depletion, even after 10 years of infection. However, the mechanisms involved in protection against HIV-1 disease progression have not been elucidated yet. Controllers represent a heterogeneous population; we describe in this paper some common characteristics concerning innate immune response and CD4+ T cells of HIV controllers.

## 1. Plasmacytoid Dendritic Cells

The innate immune system is our first line of defense against invading microorganisms while the adaptive immune system acts as a second line of defense and also affords protection against reexposure to the same pathogen. One of the hallmarks of the innate immune response is the production of the antiviral cytokine type I IFN (IFN-*α* and *β*): these factors inhibit viral replication and spreading [1]. Plasmacytoid dendritic cells (pDC) are the most potent IFN-*α*-producing cells [2, 3] and serve as an essential link between innate and adaptive immunity [4].

PDC are cells of hemopoietic origin that are found at steady state in the blood, thymus, and peripheral lymphoid tissues [5]. Early studies described pDCs as being oval shaped with typical plasmacytoid morphology. The ability of plasmacytoid-derived DCs (also named DC2) to induce a Th2 differentiation of naïve CD4+ T cells formed the basis for the concept of type 1 and type 2 DC [5]. The role of these DC in mouse and human was studied in different models and is not completely elucidated [6]. A little later, it was shown that pDC were specialized in the production of type I IFN [2]. They are the principal source of type I IFN in human blood and very rapidly produce all type I IFN isoforms in response to microbial stimuli, such as virus [2, 7], CpG-containing oligonucleotides [8], or the synthetic molecules imidazoquinolines [9]. PDC-derived type I IFN has direct antiviral activity against a variety of viruses, including HIV, and has important adjuvant functions on other immune cell types, such as NK cells, T cells, macrophages and DC [10, 11]. Thus, pDC activation triggers a dual type of response: type I IFN production and DC differentiation [5, 12].

PDCs express the Toll-like receptors TLR7 and TLR9 [13, 14] and respond to their respective ligands, imidazoquinolines [15] and single-strand RNA [16–18] for TLR7, CpG-containing oligonucleotides [19] and DNA viruses [20] for TLR9. They do not express TLR2, TLR3, and TLR4 and do not respond to such ligands as peptidoglycan, LPS (lipopolysaccharides), or double-stranded RNA [13, 14]. Activation of pDC through TLR7 and TLR9 can trigger both types of response, including large quantities of type I IFN production and/or DC differentiation [6]. Synthetic CpG-containing oligonucleotides of the types A and B (CpG-A, CpG-B) selectively induce type I IFN production and DC differentiation, respectively [21], while some viral stimuli, such as influenza virus (Flu), herpes simplex virus (HSV), or CpG-C can induce simultaneously both responses [6]. Two factors seem to be keys for the induction of large quantities of type I IFN in pDC: (1) the ability of the TLR ligands to bind its receptor in the early endosomal compartments [22, 23]; (2) the phosphorylation and nuclear translocation of the transcription factor IRF-7 [24]. This last step was shown to depend on the kinases IRAK-1 [25] and IkB kinase-a (IKK-a) [26] in mouse pDC. It has been recently shown that the PI3-kinase pathway was critical to control the nuclear translocation of IRF-7 and the subsequent production of type I IFN [27]. 

PDCs express a panel of surface receptors but pDC's function remains largely unknown. The best characterized is the lectin BDCA-2 (blood dendritic cell antigen-2) [28, 29]. Neuropilin 1 (NRP1), also called BDCA-4, is another surface receptor constitutively expressed at high levels on human pDC. NRP1 is involved in the interaction between myeloid DC and T cells within the immune synapse [30]. 

PDCs are involved in several disease settings; however, their precise role remains to be elucidated. They were observed *in situ* in a variety of pathological conditions, such as HPV-related cervical cancer, skin melanoma [31], psoriasis [32], or allergic contact dermatitis [33] and in the nasal mucosa as early as 6 hours after allergen challenge, suggesting an active recruitment of blood pDC at the site of inflammation. Moreover, a dysregulated TLR-induced IFN response has been linked to autoimmune diseases [34, 35], particularly lupus erythematous and psoriasis [32].

## 2. pDC in HIV-1 Infection

The role of pDC in HIV-1 infection is not well understood and is still debating. It may be radically different depending on the stage of the disease. The first studies reported that the number of circulating pDC was decreased in HIV-1 infection [36], and that the lack of IFN-*α* production was suggested to be responsible for HIV-1 disease progression [37, 38]. Furthermore, pDC from HIV-1-infected patients seemed to be functionally deficient as they produced less IFN-*α* in response to viral infection compared to pDC from healthy donors [39, 40]. However, recent studies highlighted a more complex role of IFN-*α* and pDC activation/death during the different stages of the disease than previously thought. It has been suggested that virus control is required to keep the balance between pDC response and pDC depletion [41]. 

The decreased number of pDC in blood could have two major causes: either pDCs undergos apoptosis due to infection by HIV-1, HIV-1 pathogenesis, and/or generalized immune dysregulation or pDCs migrate to secondary lymph tissues and potentially could die [42]. 

The first cause of pDC blood depletion could be due to the infection by HIV-1. Some arguments are in favor of this theory, as pDCs express the principal receptor for HIV-1 (CD4), but also the two coreceptors (CXCR4 and CCR5) [2]. Furthermore, it has been shown that a significant proportion of pDC was infected by SIV in a simian model [43] and that the maturation marker CD40 ligand was necessary for virus replication, which could alter pDC viability [1, 44]. Although pDCs are susceptible to HIV-1 infection and as a result may die, this is unlikely the only cause responsible for pDC depletion in humans. Indeed, approximately 1% of blood pDC from HIV-1 patients contained proviral DNA [45] and pDCs are not a major reservoir for HIV-1 *in vivo* [46]. Furthermore, we showed that infectious HIV-1, but also chemically inactivated HIV-1 (AT-2 HIV-1), had no cytotoxic effect on pDC. Actually those stimuli induced metabolic activation rather than apoptosis, due to the fact that IFN-*α* secretion provides strong antiapoptotic signals in pDC [47, 48].

The second cause of pDC depletion could be explained by migration of pDCs in lymph nodes and spleen, phenomenon that could also induce early cell death. It is likely that the second hypothesis occurs *in vivo*, with a redistribution of HIV-1-activated pDC in lymphoid organs [49]. Indeed, pDCs were found to be positive for p24 in tonsils of HIV-1-infected patients [44, 50], demonstrating an *in vivo *proof for pDC migration [51, 52]. Several studies, including ours, reported that *in vitro* HIV-1 activation of pDC resulted in cytokine production but also activation markers (CD40, HLADR), maturation markers (CD80, CD86), and migration marker CCR7 [51, 53–55]. CCR7 induces a cell migration to lymphoid organs when stimulated by its natural ligand (CCL19, CCL21). It should be noted that both infectious and noninfectious HIV-1 stimulated CCR7 expression and IFN-*α* production by pDC [56]. Furthermore, pDCs isolated from the blood of HIV-1-infected patients express CCR7 and also the activation marker HLA-DR [52]. Thus, CCR7 expression could potentially lead to migration of HIV-1-activated pDC to lymphoid organs [49]. Furthermore, Stary et al. clearly demonstrated that pDC expressed the apoptotic ligand TRAIL in viremic HIV-1-infected patients' tonsils [49]. Interestingly, in the spleens of some patients with high proviral loads, pDCs are more numerous than in controls or in spleens from patients with lower viral loads [57]. This strongly suggests a homing of pDC towards spleens in humans as well as in macaques [58].

Finally, the combination of both causes (migration and death) may explain the reduction of pDC number in the blood and lymphoid organs of viremic patients. Indeed, pDCs are lost from blood and lymph nodes two weeks after intravenous infection with HIV despite strong mobilization of pDC to lymph nodes. In lymph nodes, the vast majority of pDC is activated and only a smaller proportion undergoes apoptosis due to hostile inflammatory environment of SIV-infected macaques [59]. Furthermore, the same phenomenon of migration and depletion may occur in HIV-infected human lymph nodes [60]. 

## 3. pDC and CD4+ T Cells in HIV-Infected Controllers (HIV Controllers)

As recently suggested [41], virus control is required to keep the balance of pDC response that will result in the elimination of HIV-infected cells, reduced viral replication, and survival of CD4+ T cells. It is possible that early ART prevents chronic stimulation of the pDCs and allows the preservation of their functions, as a recent study showed that prolonged ART initiated at the time of HIV-1 seroconversion is associated with a polyfunctional immunological T-cell status that is similar to that of LTNPs [61].

Because pDC response may have a major impact for HIV-1 disease progression, studying pDC in human and simian AIDS resistant models remains essential. There is now a debate in the literature concerning the activation of pDC in AIDS-resistant simian models. One study showed that the resistance to AIDS progression in sooty mangabey model was explained by a deficient IFN-*α* production by pDC in response to SIV [62]. In contrast, it was recently shown that pDC could be activated by SIV [43] and produce high levels of type I IFN similarly in AIDS-resistant and AIDS susceptible simian models [63–65]. The kinetic study of acute and chronic SIV infection demonstrated that the difference between pathogenesis resistant African green monkey (AGM) and susceptible Rhesus macaque (RM) resides in the kinetics of interferon-stimulated genes (ISG) expression. Resistance was associated with a peak at around 2 weeks after infection, followed by a decline to near-baseline levels by 4 weeks [64, 65]. In contrast, RM followed a generally similar kinetics until the decline, with leveled off well above the baseline to become chronic [63].

In humans, the role of pDC and IFN-*α* during the different stages of HIV pathogenesis seems to be multifaceted, and it has been recently demonstrated that activated pDC and IFN-*α* contribute to chronic immune activation and T-cell depletion [49, 66]. In addition to type I IFN production, we previously showed that HIV transforms pDC into killer pDC (IKpDC) [55]. More precisely, in human tonsils, chronic activation of pDC by HIV particles induced TRAIL expression transforming them into killer cells, which induced apoptosis of CD4+ T cells [49]. Thus, we recently studied pDC response and TRAIL pathway in HIV controllers. We found that pDC from HIV controllers did not exhibit any quantitative or functional deficiencies in membrane TRAIL expression when stimulated by infectious or noninfectious HIV-1 particles. Our results also showed that *in vivo* most of pDC from HIV controllers did not express TRAIL on their membrane contrasting with the 30% of TRAIL-expressing pDC from viremic patients [39]. However, pDC from controller patients showed high levels of membrane TRAIL expression after viral stimulation. In addition, microscopy analysis of circulating pDC from viremic patients revealed the existence of TRAIL expressing IKpDC [39], confirming the *in vivo *generation of this cell subset during HIV infection [49].

**Figure 1:**
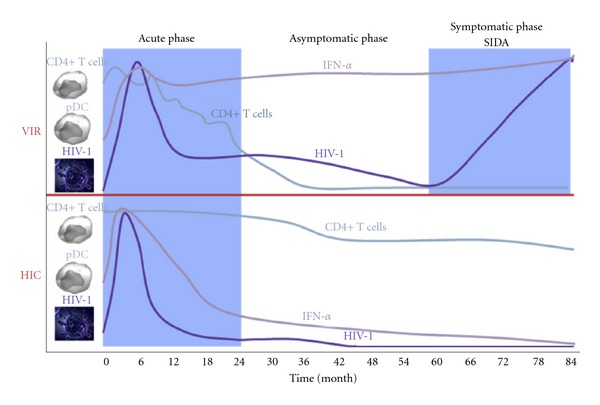
Comparison between HIV-1 viremic patients and HIV-1-Infected controllers during the different phases of infection. Viral load and lymphopenia controlled in HIV controllers versus viral load and lymphopenia uncontrolled in viremic. Decrease of IFN-*α* production versus no decrease of IFN-*α* production in viremic.

Thus, PDCs from HIV controllers produce IFN-*α* and express membrane TRAIL after viral stimulation *in vitro*, transforming them into IKpDC [39, 40]. The fact that pDCs from HIV controllers do express mTRAIL after stimulation suggests that they are less stimulated *in vivo,* probably due to the nearly absence of HIV particles in the blood. Thus, pDCs are not activated and probably do not migrate to lymphoid organs, explaining the elevated number of pDC in blood from HIV controllers compared to viremic patients. These results are consistent with our previous study showing that tonsils from controllers do not exhibit IFN-*α* staining and TRAIL mRNA in contrast to tonsils from viremic [52]. In conclusion, qualitative and functional involvement of pDC is necessary for the spontaneous control of HIV viremia. These findings highlight the important role of innate immunity in HIV immunopathogenesis and could have important immunotherapeutic applications. Furthermore, in accordance to simian models, HIV controller patients maintained elevated pDC blood number. In culture, pDCs from HIV controller produce high levels of IFN-*α* in response to HIV exposure contrasting with pDC from viremic patients that statistically produced less IFN-*α* than pDC from HIV controllers (Figure 1 and Table 1).

Finally, we observed that IKpDC could induce apoptosis of CD4+ T cells. This result combined with TRAIL expression and the high levels of IFN-*α* produced by pDC from HIV controllers after viral stimulation suggest that, in contrast to some simian models, pDCs from controllers are fully functional and do not show any impairment. However, HIV controllers usually keep high CD4+ T-cell counts (Figure 1). Interestingly, we found that CD4+ T cells from HIV controllers did undergo only limited apoptosis when cultured with HIV (personal data). In contrast, in our study, we clearly demonstrated that pDCs from HIV controllers are able to induce apoptosis of DR5 expressing cell line, thus this finding can not be explained by a weak pDC functionality in HIV controllers but by a particularity of CD4+ T cells that remained to be investigated. 

CD4+ T cells from elite controllers are less susceptible to HIV infection compared to HIV progressors and healthy donors. HIV gene expression is decreased when the known tumor suppressor gene called p21 is upregulated as observed in most of HIV controllers. This potentially leads to the resistance of those controllers. 

Multiple mechanisms contribute to CD4+ T-cell depletion in HIV infection (reviewed in [67]). During chronic immune activation, there is an increase programmed cell death (apoptosis) of CD4+ T cell subsets. The decline of the rate of memory CD4+ T cells (T_CM_ cells) is predictive for disease progression. The resistance of those cells to apoptosis correlates with immunological protection in HIV and SIV infections [68]. We then wonder whether CD4+ T cells in HIV controllers have a higher survival rate compared to subjects with progressive disease. A recent study [69] showed that when T-cell receptors are triggered, T_CM_ and effector memory CD4+ T cells (T_EM_ cells) from elite controllers are less susceptible to Fas-mediated apoptosis and persist longer compared to CD4+ T cells from HAART patients and from healthy donors. 

The hallmark of HIV infection is CD4+ T-cell depletion. This lymphopenia is due to several combined effects: destruction of HIV-infected cells, increased cell death of uninfected CD4+ T cells, and impair renewal [70]. Functional memory T cells have a long-lasting protection after exposure to pathogens [71, 72]. Preservation of the central memory (T_CM_) CD4+ T-cell compartment observed in HIV controllers was better than in untreated HIV progressors and individuals under therapy [73]. We also noticed a higher expression of the IL-7 receptor and CCR7, which suggests differences in T_CM_ of HIV controllers homing patterns [73].

Physiological signals, in particular to immunomodulatory cytokines, decrease during chronic immune phase of HIV infection. One of the first signs of the immune deficiency that precedes CD4+ lymphopenia in HIV-infected patients is the reduction of IL-2 production and the lack of response to this cytokine [74–77]. Furthermore, it has been shown that progressive HIV disease is associated with a decrease in responsiveness to IL-7 [78, 79], a cytokine that is crucial to the central production of CD4+ lymphocytes and to their peripheral homeostasis (reviewed in [80]). The T-cell access to IL-7 on the fibroblastic reticular cell network is restricted by collagen deposition in lymphoid tissue, resulting in apoptosis and depletion of T cells [81]. Two clinical trials using IL-7 administration in ART-treated patients gave promising results in terms of reconstitution of CD4+ T-cell subsets [82, 83]. IL-7 treatment may also help to purge viral reservoirs [80].

HIV-specific CD4+ T cells from HIV controllers divide more rapidly than those from viremic patients or ART patients [84]. HIV-specific CD4+ T cells in HIV controllers have the capacity to respond to minimal amounts of antigens. This may contribute to the better response of HIV-specific CD4+ T cell observed in controllers compared to ART-treated subjects. It remains to be determined whether or not HIV-specific CD4+ T cells can also have significant antiviral effects *in vivo*.

During acute HIV infection, massive destruction of the memory CD4+ T-cell compartment mostly occurs in lymphoid tissues, in particular in the gut-associated lymphoid tissue (GALT) [67, 85, 86]. All these events are mainly responsible for HIV immunopathogenesis. We noticed that the level of T-cell activation is lower in HIV controllers than in untreated viremic subjects, but higher than observed in healthy donors and individuals on successful ART [87]. Indeed, data indicate the presence of bacterial translocation in HIV controllers [87], in contrast to healthy donors. This indicates that HIV controllers have a successful viral control and they present lower detrimental consequences of chronic immune activation, including CD4+ T-cell loss [87]. 

In conclusion, HIV-specific CD4+ T cells are associations of some HLA Class II alleles with lower viral loads showed in some reports, which has so far not been confirmed in GWAS studies of HIV controllers. CD4+ T cells from HIV controllers are also more robust and polyfunctional in terms of cytokine secretion. They maintain high responses in spite of low antigen load, in contrast to ART-treated subjects in whom HIV-specific CD4+ T cells decrease on therapy. There is a higher growth kinetics and functional avidity of HIV-specific CD4+ T cells of HIV controllers. Interestingly, we noticed a lower expression of CTLA-4, an important inhibitory molecule, on HIV-specific CD4+ T cells of elite controllers.

## 4. Hypothetic Model for HIV Controllers

Recent findings concerning HIV controllers argue in favor of a strong pDC response during acute infection and a significantly more robust, polyfunctional HIV-specific CD4+ T cells. 

The first response against HIV infection involves IFN-*α* production by most of immune cells including pDC. The fact that IFN-*α* production was higher in HIV controllers than in viremic patients suggests that the control of HIV replication could be better (Figure 1) [40]. In addition to this efficient viral control, HIV-specific CD4+ T-cell response was shown to have a higher magnitude. HIV-specific CD4+ T cells from HIV controllers divide more rapidly than comparable lines from viremic patients. This study also identified a subpopulation of HIV-specific CD4+ T cells in HIV controllers endowed with a higher functional avidity compared to noncontrollers. 

Considering these experimental data, we can hypothesize that innate immune response during acute phase is central and will determine the controller phenotype. Increased IFN-*α* production and better CD4+ T-cell response will lead to an efficient viral control. In our hypothetic model, the number of viruses is crucial. We demonstrated that the apoptotic marker TRAIL, which is regulated by type I IFN, was positively correlated to viral load. Because TRAIL is probably one of the molecules responsible for T-cell depletion, the less the TRAIL is produced the less the disease progression is important. Similarly, during chronic phase, a reduced number of HIV particles induce less IFN-*α* production, less proinflammatory cytokines. Thus, the chronic immune activation, which is probably one the most important processes leading to lymphopenia, is reduced in HIV controllers.

## Figures and Tables

**Table 1:** Characteristic of HIV controllers and viremic patients.

Group	Age	Viral load (copies/mL)	CD4+ count (cells/mL)	Blood pDC number	IFN-*α* production	*In * *vivo* TRAIL
Controllers (*n* = 22)	45	<50	718	No decrease	++++	No
Viremic (*N* = 18)	42	26700	612	Decrease	+	Yes

## Abbreviations

(IFN-*α*): Interferon alpha
(TLR): Toll-like receptor
(pDC): Plasmacytoid dendritic cell
(TRAIL): TNF-related apoptosis-inducing ligand
(IKpDC): TRAIL-expressing interferon-producing killer pDC
(DR5): Death Receptor 5
HIV-1: Human immunodeficiency virus type 1.
